# Appendicitis and Pseudo-Appendicitis in Certain Infectious Diseases.—II

**Published:** 1911-06-10

**Authors:** 

**Affiliations:** Physician to the Hospital "Des Enfants Malades," Professor in the Faculty of Médecine, Member of the Academy of Médecine, Paris.


### Hospital Clinics.

### APPENDICITIS AND PSEUDO-APPENDICITIS IN CERTAIN INFECTIOUS DISEASES.—II.

A Lecture by Dr. HUTINEL, Physician to the Hospital " Des Enfants Malades," Professor in the Faculty of Médecine, Member of the Academy of Médecine, Paris.

A few weeks ago we had a similar case in the " Bouchut " ward. A big boy of 14 came to the hospital for pain distinctly localised in the right iliac fossa; the abdomen was distended, temperature 104° F., pulse extremely rapid, vomiting and constipation. Appendicitis was diagnosed, but five or six days later rose-spots appeared, the appendicular symptoms diminished, the fever fell, and a positive sero-diagnosis was obtained by Dr. Nobecourt. It was a case of enteric with appendicular onset. Similar cases have been noted by Drs. Moizard, Mery, Comby, Barbier, and Broca, and were the subject of very interesting discussions at the Society of Pediatry.

### No General Rule for Operation.

As, in these cases, surgical intervention is always useless and sometimes dangerous (Dr. Barbier's case ended fatally) it is very important to make an early diagnosis. Examine your patients most minutely, take into careful consideration the indications furnished by the pulse and temperature, explore the abdomen very carefully, ascertain whether the spleen is enlarged or not. In Dr. Bayet's opinion the pain in the right fossa is more diffused in enteric and more localised in appendicitis. The same author advised systematic operation in all cases; personally I prefer abstention, but do not ask me for any general rule. In my opinion there are different forms of appendicitis—each presents special indications, and it is impossible to formulate any general precepts embracing all cases; we must show ourselves opportunists as the occasion demands.

### Purpura.

I was speaking to you only last week of *purpura* and I told you that in one of its forms, the " rheumatoid form," it was quite frequent to note abdominal pain. I have long ago described cases in which purpura simulates lead colic, intestinal strangulation, or appendicitis, and which end favourably after the evacuation of bloody stools. Dr. Calmets in his thesis, " Les troubles gastro-intestinaux du purpura," and quite recently Dr. Vieillard, a pupil of Dr. Guinon, have described similar cases. When in the course of rheumatoid purpura you observe appendicular symptoms, remember that the purpuric eruption may take place on the peritoneum of the right fossa or on the appendix, and do not be in too great a hurry to advise operation. Dr. Ardin-Delteil has described in the " Montpelier Medical," 1905, the case of a woman of twenty-seven suffering from rheumatoid purpura who, five days after the beginning of the trouble vomited black matter, passed blood-stained stools, presented multiple hæmorrhages, and finally died. He considered this a case of masked appendicitis having determined diverse hæmorrhages and amongst others purpuric spots. He concludes that whenever one is in presence of a case of purpura the abdomen should be explored carefully, particularly the region of the appendix.

### A Case in Point.

I have seen a very analogous case. A patient presented first of all very serious gastro-intestinal symptoms, then purpura, and a little later on the symptoms localised in the right fossa; the patient died. I wondered then, and do so now, whether, in this case, the infection was primarily and purely appendicular or whether the lesions of the whole intestinal tract did not contribute as much as those of the appendix in producing the symptoms noted. Three years ago Dr. Jalaguier operated on a young girl of twelve suffering from appendicitis; the organ was very large and turgescent. The operation was perfectly successful, but a few days later the child presented marked pallor and intestinal hæmorrhage, hæmoptysis, and purpura supervened. In order to combat the anæmia, radiotherapy was applied and seemed to give good results during a few weeks, but the hæmorrhages soon reappeared and the patient died. It was a case of pernicious anæmia with appendicular onset.

I wish now to say a few words on the connection between appendicitis and certain affections of the respiratory organs: pleurisy, pulmonary gangrene, and especially pneumonia. About twelve years ago or so I observed the following case: A little girl presented suddenly all the symptoms of appendicitis; pain at McBurney's point, fulness in the right fossa, vomiting, constipation, and fever. She was operated and the surgeon to his great surprise found the appendix absolutely healthy. Two days later the temperature fell suddenly from 104° to 98°; it turned out to be a case of pneumonia.

### Another Case.

An Englishman, after a drinking bout, was taken suddenly with pain in the right fossa, vomiting, and fever. A physician was called, diagnosed appendicitis, and called in my friend, Dr. Quenu, to perform the operation. Before the latter was undertaken, however, I was asked to give my opinion on the case. On examination I found tubular breathing and fine crepitant râles at the right base; the patient was suffering from pneumonia, and it is needless to say he was not operated upon. He died a few days later, thus justifying the generally accepted opinion of the extreme gravity of pneumonia in alcoholic subjects. I should never finish if I were to describe all the analogous cases; they have been studied by Drs. Miraude, Hazard, and Gaveau in their theses.

Allow me, however, to recall a case that I saw with Dr. Darré. A boy of eight was taken suddenly with bilious vomiting and pain in the right fossa; the temperature went up to 104°, the pulse to 130, but as there was no swelling in the region of McBurney's point, and as the patient presented marked dyspnoea, I diagnosed pneumonia. My friend Jalaguier, who was called in, refused at first to pronounce himself, but thought that my diagnosis might turn out correct. As a matter of fact two days later a typical defervescence took place.

You are aware that pneumococcic infection may affect the peritoneum; the cases described by Duparque (1842), Fereol (1859), Rillet and Barthez (1861), Bauver, Gauderon (1876), and the more recent works of Netter, Sevestre, Gaillard, and Michaux have settled the question of pneumo-coccic peritonitis. The affection may present two forms, the diffused and the encysted forms. It is more frequent in girls and may be secondary to some other localisation of pneumonia, although in most cases it is primary. The pathogeny has given rise to much discussion for the peritoneal infection may take place through different channels: lymphatic, blood, genital, intestinal, and even perhaps appendicular. However this may be, it causes abdominal pain, vomiting, and diarrhœa, then the pain diminishes and the vomiting ceases, but the abdomen becomes distended and pus forms. In certain cases it is not at all easy to distinguish this pneumo-coccic peritonitis from appendicitis. I saw some little time ago a girl who was taken suddenly with fever, vomiting, and diarrhœa. Pain soon appeared and localised in the right fossa with irradiations towards the iliac bones; palpation showed the presence of a deep-seated purulent collection; the temperature presented marked oscillations. I diagnosed peri-appendicular suppuration of pneumo-coccic origin. Operation confirmed the diagnosis, and a microscopic examination showed the presence of pneumo-cocci in the pus. The patient recovered.

What conclusion can we draw from all the cases I have described to you except that the appendix is, of all the different regions of the intestines, the one which is the most easily infected. Whenever a general infection takes place (scarlet fever, measles, influenza, pneumonia) or a local infection exists (entero-colitis, peritonitis, etc.) the appendix is very liable to be affected also, especially if it has already been damaged. You must bear in mind, therefore, the possibility of appendicular reaction and that the same treatment is not applicable in all cases. In some simple medical treatment is sufficient; in others early surgical intervention is necessary. Each case must be judged on its own merits for, as I have already said, there are no absolute laws in medicine and only the closest study of your patients can be your guide.

